# Effects of face masks on performance and cardiorespiratory response in well-trained athletes

**DOI:** 10.1007/s00392-021-01877-0

**Published:** 2021-06-06

**Authors:** Florian Egger, Dominic Blumenauer, Patrick Fischer, Andreas Venhorst, Saarraaken Kulenthiran, Yvonne Bewarder, Angela Zimmer, Michael Böhm, Tim Meyer, Felix Mahfoud

**Affiliations:** 1 Department of Internal Medicine, Saarland University Hospital, Homburg, Saar Germany; 2 Institute of Sports and Preventive Medicine, Saarland University, Campus, Building B8 2, 66123 Saarbrücken, Germany

**Keywords:** Masks, Cardiopulmonary exercise test, Athletes

## Abstract

**Background:**

During the COVID-19 pandemic, compulsory masks became an integral part of outdoor sports such as jogging in crowded areas (e.g. city parks) as well as indoor sports in gyms and sports centers. This study, therefore, aimed to investigate the effects of medical face masks on performance and cardiorespiratory parameters in athletes.

**Methods:**

In a randomized, cross-over design, 16 well-trained athletes (age 27 ± 7 years, peak oxygen consumption 56.2 ± 5.6 ml kg^−1^ min^−1^, maximum performance 5.1 ± 0.5 Watt kg^−1^) underwent three stepwise incremental exercise tests to exhaustion without mask (NM), with surgical mask (SM) and FFP2 mask (FFP2). Cardiorespiratory and metabolic responses were monitored by spiroergometry and blood lactate (BLa) testing throughout the tests.

**Results:**

There was a large effect of masks on performance with a significant reduction of maximum performance with SM (355 ± 41 Watt) and FFP2 (364 ± 43 Watt) compared to NM (377 ± 40 Watt), respectively (*p* < 0.001; *ηp*^2^ = 0.50). A large interaction effect with a reduction of both oxygen consumption (*p* < 0.001; *ηp*^2^ = 0.34) and minute ventilation (*p* < 0.001; *ηp*^2^ = 0.39) was observed. At the termination of the test with SM 11 of 16 subjects reported acute dyspnea from the suction of the wet and deformed mask. No difference in performance was observed at the individual anaerobic threshold (*p* = 0.90).

**Conclusion:**

Both SM and to a lesser extent FFP2 were associated with reduced maximum performance, minute ventilation, and oxygen consumption. For strenuous anaerobic exercise, an FFP2 mask may be preferred over an SM.

## Introduction

Face masks have been shown to actively contain transmission of SARS-CoV-2 [1–3]. During the COVID-19 pandemic, public authorities in various countries made it mandatory to wear face masks in indoor and outdoor public places. Additionally, compulsory masks became an integral part of sport including gyms and sports centers. However, uniform recommendations for athletes to wear a face mask during exercise do not yet exist. According to the United States Center for Disease Control and Prevention guidelines, masks are especially recommended especially for indoor sports such as basketball, but also for low- and high-intensity outdoor training whenever feasible [4]. The World Health Organization has published less precise recommendations and advised against wearing masks during strenuous exercise [5]. Exercising at higher intensities might lead to increased moisture retention resulting in the deformation of less rigid masks [6]. A wet mask is harder to breathe through and filters less efficiently and it has therefore been recommended to change masks regularly when exercising [4]. To date, few studies investigated the effect of face masks on physical performance in healthy (untrained) individuals [7–10].

Data on the effects of face masks during strenuous exercise are scarce, with a single study focusing on steady-state exercise [10]. To the best of our knowledge, no study has yet examined the effects of face masks on the individual anaerobic threshold in athletes, an intensity marker commonly used for training prescriptions in endurance sports [11, 12]. This study, therefore, aimed to investigate the effects of medical face masks on both maximal and submaximal performance in well-trained athletes regularly participating in training and competition, to provide evidence for training recommendations including preventive aspects as potential side effects of masks.

## Methods

### General design

In a randomized, cross-over design, subjects underwent three stepwise incremental exercise tests: (i) one without mask (NM), (ii) with a surgical mask (SM) and (iii) with a FFP2 mask (FFP2). All tests were performed at the same time of day, at least 48 h apart from each other, and completed within two weeks. During the test period, subjects were instructed to continue their normal training routine but to abstain from strenuous and prolonged (> 30 min) exercise 24 h prior to the assessment and to maintain their usual carbohydrate-rich diet. Prior to each test, subjects received standardized questions regarding self-evaluation of physical condition, hydration status, and compliance to dietary and exercise restrictions. All tests were carried out under standardised laboratory conditions with measurement of relative humidity and ambient temperature (Sonomo thermo- and hygrometer, Wuhan, China; measurement interval 10 s).

### Subjects

Sixteen well-trained, healthy male athletes (2 road cyclists, 8 mountain bikers, 6 triathletes; peal oxygen consumption (*V*O_2peak_) 56.2 ± 5.6 ml kg^−1^ min^−1^, age 27 ± 7 years, BMI 22.5 ± 1.8 kg m^−2^) volunteered to participate in this study. On their first visit to the laboratory, each subject underwent a medical check-up consisting of a physical examination, history, 12-lead ECG, and resting office blood pressure measurement. Further investigations (blood count, whole-body plethysmography, echocardiography) were carried out only if medically indicated. Subjects were eligible if they met the following inclusion criteria: age 18–40 years, cycle training ≥ 6 h per week, individual anaerobic threshold (IAT) > 200 Watt (W), maximum performance during a stepwise incremental cycle test of at least 4.6 W kg^−1^ (performance level 3) [13]. All subjects were fully informed about the experimental procedures and provided written informed consent prior to participation. The study was carried out in accordance with the declaration of Helsinki and approved by the local ethics committee (Ärztekammer des Saarlandes, Saarbrücken, Germany; approval number: 199/20).

### Incremental exercise test

Subjects performed all trials with clipless pedals on their own bikes attached to an electromagnetically bicycle ergometer (Cyclus 2, RBM elektronik-automation GmbH, Leipzig, Germany). Individual seat and handlebar position was maintained throughout. Subjects started cycling at 100 W or 150 W and workload was increased every 3 min by 50 W until exhaustion. After each step subjects were asked to rate their rate of perceived exertion (RPE) on a 10-point scale (Borg CR10) [14]. Arterialized capillary blood was obtained from the hyperemised earlobe for analyzing blood lactate (BLa; enzymatic-amperometric method, Greiner, Flacht, Germany) at rest, at the end of each step, at cessation and 1, 3, 5, 7 and 10 min post-exercise. Based on a performance curve, IAT was determined using the methods described by Stegmann et al. [15]. Tests were terminated when subjects signalized maximum exhaustion or were unable to maintain a pedaling cadence of 50 revolutions per minute (rpm) for more than 10 s. Objective criteria for exhaustion (i.e. heart rate (HR)_max_ [208 – 0.7 × age]; BLa_max_ > 9 mmol l^−1^; respiratory quotient > 1.1) were applied according to the current literature [16, 17]. Gas exchange parameters were measured continuously with a MetaMax II metabolic test system (Cortex Biophysik, Leipzig, Germany; mixing chamber; sampling frequency 10 s). Time courses of oxygen consumption (*V*O_2_) and respiratory minute ventilation (VE) were calculated using the mean of 3 contiguous values at the end of each step and at exhaustion. Heart rate was derived from continuous 12-lead ECG recordings. Blood pressure was measured manually at rest, at the end of each step, and 1, 3, 5 min post-exercise.

### Fitting of the face masks

FFP2 protective face masks (Shezhen Source Innovation Technologies Co. Ltd., Guangdong, China) and surgical masks (Quanzhou Nanfang Sanitary Products Co. Ltd., Quanzhou City, China) were used for this study. A loose spirometry mask (not connected to the volume sensor) was placed over the respective face mask and fixed and tightened with head straps. Subsequently, the subject was asked to close the valve of the spirometry mask with the heel of his hand and to exhale firmly against it to check for leaks. This maneuver was repeated under the careful supervision of the examiner until no acoustic, visual, and sensory indications of leakage were detectable. By selecting 3 different mask sizes (small, medium, large) an adequate seal of the mask could always be achieved before attaching the volume sensor and starting the measurement.

### Outcome measures

Maximal performance (*P*_max_) and submaximal performance at IAT (*P*_IAT_) were the main outcome measures in this study. Secondary outcome measures included HR, blood pressure, VE, *V*O_2_, carbon dioxide production (*V*CO_2_), BLa, and RPE.

### Statistical analysis

Since there were very limited data published to establish baseline assumptions, no formal sample size estimation was performed. Subjects were randomly assigned to the sequence of exercise with NM, SM or FFP2. Statistical analyses were performed using GraphPad (version 9.0).

Data were screened for outliers, in one case multiple imputation procedure was applied. Because all dependent variables were normally distributed (Shapiro–Wilk test), parametric tests were applied, and data are presented as mean ± standard deviation. Differences between conditions were tested for significance using one-factorial ANOVA. Differences in time courses of HR, BLa, *V*O_2,_ and VE between conditions during incremental exercise tests were compared using two-way repeated-measures ANOVA. Greenhouse–Geisser epsilon adjustment was made when sphericity was violated. When the difference between means was significant, post-hoc comparisons were performed by means of the Tukey test. Effect sizes were calculated as partial eta-squared (*ηp*^2^) and interpreted as small (0.01), medium (0.06), or large (0.14) [18].The significance level for the alpha error was set at *p* < 0.05.

## Results

All 16 subjects completed the study and the three incremental exercise tests were performed within 9 ± 4 days (range 5–14 days) between June 2020 and January 2021. There was no difference in ambient temperature (NM: 21.6 ± 2.6 °C; SM: 20.3 ± 3.6 °C; FFP2: 21.7 ± 2.1 °C; *p* = 0.34) and relative humidity (NM: 38.4 ± 5.4%; SM: 37.9 ± 6.0%; FFP2: 40.3 ± 4.9%; *p* = 0.21) during the incremental exercise tests. For each condition, at least one of the objective criteria for exhaustion was exceeded.

### Performance

**Fig. 1:**
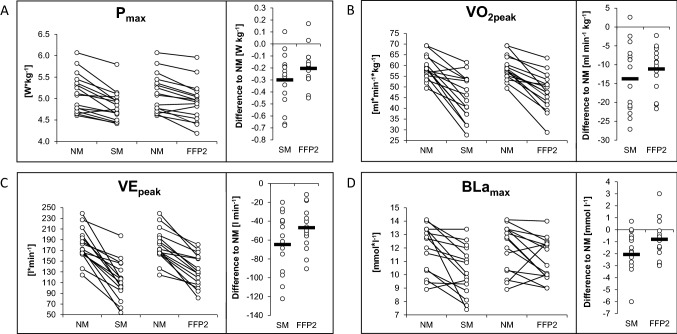
Interindividual differences at exhaustion during the incremental exercise test (*n* = 16) without a mask (NM), with a surgical mask (SM), and with FFP2-mask (FFP2). **A** maximum performance (*P*_max_). **B** peak oxygen consumption (*V*O_2peak_). **C** peak minute ventilation (VE_peak_), **D** maximum blood lactate concentration (BLa_max_). *W* Watt

*P*_max_ differed significantly between NM (377 ± 40 W), SM (355 ± 41 W) and FFP2 (364 ± 43 W), respectively (*F*_2,30_ = 18.3; *p* < 0.001; *ηp*^2^ = 0.5). Compared with NM, *P*_max_ was decreased by 21.3 ± 15.1 W [95% confidence interval (CI) 11.5 to 31.1, *p* < 0.001] and 12.4 ± 13.2 W (95% CI 3.9 to 21.0, *p* < 0.01) with SM and FFP2, respectively. The reduction of *P*_max_ compared with NM was − 5.6 ± 4.1% (95% CI − 3.5 to − 7.8, *p* < 0.001) and − 3.7 ± 3.1% (95% CI − 2.2 to − 5.7, *p* < 0.01) with SM and FFP2, respectively. The difference of 8.8 ± 14.0 W in *P*_max_ between SM and FFP2 did not reach statistical significance (95% CI − 18.0 to 0.3, *p* = 0.06). *P*_max_ related to body weight was decreased by − 0.3 ± 0.2 W kg^−1^ (95% CI − 0.4 to − 0.2, *p* < 0.001) and − 0.2 ± 0.1 W kg^−1^ (95% CI − 0.3 to − 0.1, *p* < 0.001) with SM and FFP2 compared to NM, respectively (Fig. 1). No difference in *P*_IAT_ was observed between conditions (*F*_1.7;25.2_ = 0.07; *p* = 0.90).

### Cardiocirculatory, respiratory and metabolic parameters

**Fig. 2:**
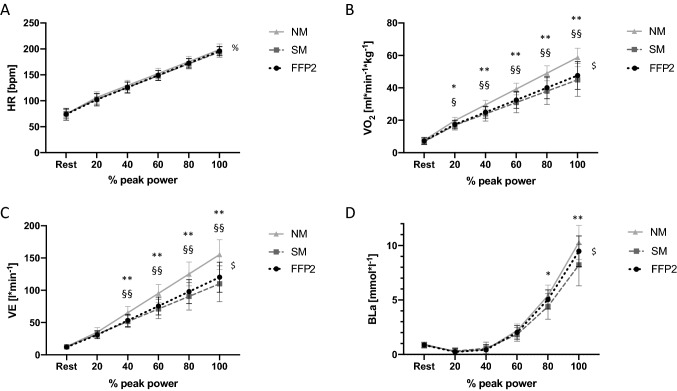
Mean changes in physiological parameters throughout the incremental exercise test (*n* = 16) without a mask (NM), with a surgical mask (SM), and with FFP2-mask (FFP2). **A** Heart rate (HR) in beats*min^−1^ (bpm). **B** oxygen consumption (*V*O_2_) in ml kg^−1^ min^−1^. **C** minute ventilation (VE) in l min^−1^. **D** blood lactate concentration (BLa) in mmol l^−1^. Error bars represent standard deviation. *p < 0.05, NM vs. SM. **p < 0.01, NM vs. SM. ^§^*p* < 0.05, NM vs. FFP2. ^§§^*p* < 0.01, NM vs. FFP2. % = time effect. $ = interaction effect

Time courses of HR, *V*O_2_, VE and BLa as a function of performance (percentage of peak power output) are illustrated in Fig. 2. HR did not differ between conditions at any scalar time point during the incremental cycling test (*F*_10,225_ = 1.0; *p* = 0.37). There was a large interaction effect for both *V*O_2_ (*F*_10,225_ = 11.8; *p* < 0.001; *ηp*^2^ = 0.34) and VE (*F*_10,225_ = 14.6; *p* < 0.001; *ηp*^2^ = 0.39) between conditions over time showing decreases for both SM and FFP2 compared with NM at 40%, 60%, 80% and 100% of peak power output. The change in V*O*_2peak_ was − 13.8 ± 9.0 ml kg^−1^ min^−1^ (95% CI − 19.6 to − 7.9, *p* < 0.001) and − 11.1 ± 5.9 ml kg^−1^ min^−1^ (95% CI − 15.0 to − 7.3, *p* < 0.001) and the change in peak minute ventilation was − 64.7 ± 33.0 l min^−1^ (95% CI − 86.1 to − 43.2, *p* < 0.001) and − 46.9 ± 22.5 l min^−1^ (95% CI − 61.5 to − 32.3, *p* < 0.001) with SM and FFP2, respectively (Fig. 1).

Moreover, a large interaction effect for BLa between conditions and time of measurement (*F*_10,210_ = 5.5; *p* < 0.001; *ηp*^2^ = 0.2) was present and post-hoc tests located a decrease in BLa concentration by 1.0 mmol l^−1^ at 80% (95% CI 0.04 to 1.0, *p* = 0.04) and by 2.0 mmol l^−1^ at 100% of peak power output (95% CI 0.5 to 3.5, *p* = 0.008) between SM and NM, respectively (Fig. 2). With FFP2 the decrease of BLa_max_ by − 0.8 mmol l^−1^ (95% CI − 1.9 to 0.24, *p* = 0.14) was not significant (Fig. 1). The exercise-dependent responses of additional parameters such as blood pressure, HR recovery and *V*CO_2_ between conditions are presented in Table 1.

**Table 1:** Cardiocirculatory, respiratory and metabolic parameters at rest, during and after an incremental cycling test

Incremental cycling test	Unit	NM	SM	FFP2	ANOVA	NM vs. SM	NM vs. FFP2	SM vs. FFP2
Rest								
Heart rate	bpm	74 ± 9	75 ± 8	74 ± 11	0.850	–	–	–
Systolic blood pressure	mmHg	128 ± 10	128 ± 12	130 ± 12	0.790	–	–	–
Blood lactate	mmol l^-1^	0.9 ± 0.2	0.8 ± 0.2	0.9 ± 0.2	0.380	–	–	–
*V*O_2_	(ml/min)/kg	7.7 ± 1.6	6.8 ± 1.8	7.2 ± 1.7	0.130	–	–	–
*V*CO_2_	(ml/min)/kg	7.3 ± 1.3	6.5 ± 1.8	6.9 ± 2.1	0.250	–	–	–
VE	l/min	12.7 ± 2.0	12.5 ± 1.9	11.9 ± 1.3	0.200	–	–	–
IAT								
Performance	W/kg	3.7 ± 0.5	3.7 ± 0.3	3.7 ± 0.4	0.900	–	–	–
Heart rate	bpm	170 ± 13	169 ± 14	169 ± 12	0.770	–	-	–
Blood lactate	mmol/l	3.0 ± 0.4	2.8 ± 0.6	2.9 ± 0.6	0.490	–	-	–
Exhaustion								
Performance	W/kg	5.1 ± 0.5	4.8 ± 0.4	4.9 ± 0.5	** < 0.001**	** < 0.001**	** < 0.001**	–
Heart rate	bpm	191 ± 9	189 ± 9	191 ± 7	0.290	–	–	–
Systolic blood pressure	mmHg	227 ± 15	223 ± 17	226 ± 16	0.570	–	–	–
Blood lactate		11.9 ± 1.8	10.3 ± 1.8	11.1 ± 1.6	**0.003**	**0.005**	–	–
* V*O_2_	(ml/min)/kg	58.8 ± 5.7	45.0 ± 10.2	47.6 ± 8.5	** < 0.001**	** < 0.001**	** < 0.001**	–
*V*CO_2_	(ml/min)/kg	70.6 ± 7.7	49.7 ± 13.8	56.3 ± 12.4	** < 0.001**	** < 0.001**	** < 0.001**	–
VE	l/min	179.4 ± 30.2	114.7 ± 36.3	132.5 ± 29.8	** < 0.001**	** < 0.001**	** < 0.001**	–
Post-exercise								–
HRR 1 min post	bpm	− 41 ± 10	− 36 ± 12	− 42 ± 13	0.170	–	–	–
HRR 3 min post	bpm	− 69 ± 7	− 72 ± 11	−74 ± 10	0.120	–	–	–
HRR 5 min post	bpm	− 78 ± 9	− 81 ± 11	− 81 ± 10	0.150	–	–	–

Significant results are presented in bold. *V*O_2_ oxygen uptake, *V*CO_2_ carbon dioxide production, *VE* minute ventilation, *IAT* individual anaerobic threshold, *HRR* heart rate recovery, *bpm* beats per minute, *W* Watt

### Subjective criteria for exhaustion

Exercising without a mask was terminated by all subjects due to volitional exhaustion (*n* = 16). No difference between conditions was observed for RPE throughout the incremental cycling test (*F*_10, 225_ = 0.4; *p* = 0.93). At higher intensities with SM, all 16 subjects uniformly reported increasing wetting and deformation of the mask, which was also observed by the investigators when removing the spirometry mask post-exercise. Acute dyspnea due to suction of the mask led to abrupt exercise cessation in 11 of 16 subjects (69%) with SM. With FFP2, none of the subjects reported mask deformation and only 2 of 16 subjects (13%) complained of moisture retention within the mask at higher intensities.

## Discussion

This randomized cross-over study was the first to investigate the effects of medical face masks on performance in well-trained athletes. Firstly, wearing a face mask was associated with a significant reduction (almost 6%) in maximum power output (more pronounced for SM than FFP2), but not associated with a change in submaximal performance (workload at IAT). Secondly, during the incremental cycling tests, *V*O_2_ and VE were reduced with both masks and BLa with SM. Thirdly, no difference in RPE was observed between conditions during exercise, however, subjects reported a considerable rate of abrupt exercise cessation due to acute dyspnea associated with suction of the wet and deformed mask (SM), or to a lesser extent, discomfort at higher intensities from moisture retention alone (FFP2).

### Performance

Our results are supported by a recent study in healthy volunteers showing that wearing an FFP2 mask (and to a lesser extent SM) was associated with reduced *P*_max_, *V*O_2_ and VE during an incremental exercise test [9]. As in the present study, the underlying physiological effect for performance impairment appears to be pulmonary, due to reduced VE indicating increased breathing resistance of the mask, which has already been shown for respiratory protective devices, externally added breathing resistance or N95 respirators [19–21]. Moreover, a recent study found a two-fold higher airway resistance with SM compared to NM [10]. However, two previous studies found no detrimental effects on maximum cycling performance [7, 8]. When comparing SM, N95 respirators, and NM, cycling time to exhaustion did not differ in sixteen untrained males, and no symptoms emerged despite a mild increase in end-tidal carbon dioxide (re-breathing of the expired air) with both masks [7]. Furthermore, there was no difference in performance, tissue oxygenation index or arterial oxygen saturation between SM, cloth masks or NM in fourteen untrained volunteers [8]. In these studies, however, the detrimental effect of (surgical) masks on performance (soaking and deformation of the mask associated with acute dyspnea) was not present as observed in our study. In contrast to our study, the study population consisted of untrained individuals or recreational athletes (non-cyclists) who typically do not reach physical exhaustion (early test termination due to leg fatigue, poor motivation or decreased pain tolerance) compared with well-trained cyclists [22, 23]. Therefore, SM soaking and deformation probably did not occur to the same extent as in the endurance cyclists studied herein, as moisture retention increases with exercise intensity (proportion of oral breathing) and sweating [24, 25]. In addition, athletes achieve higher peak minute ventilation due to their higher cardiopulmonary exercise capacity compared to untrained individuals, which may explain the increased moisture retention. The adverse effect on performance with SM (and to a lesser extent with FFP2) in our study could at least partly be due to increased breathing resistance, which has been shown to be associated with moisture retention [26].

With FFP2, our subjects reported no mask deformation or suction associated with acute dyspnea, most likely because this mask type is more rigid than SM [6]. In our study, the performance impairment with both masks occurred acutely and to some extent unexpectedly, which is supported by the unchanged RPE between all conditions. In this context, an equally high RPE value with absolutely lower performance with SM and FFP2 corresponds to a relative RPE increase. The lower BLa concentrations at the highest intensity with FFP2 and SM, as observed recently [9], appear to be a reflection of early exercise cessation rather than a consequence of wearing a mask.

### Physiological response

This study was the first to estimate the effects of wearing a face mask on submaximal performance in athletes, more specifically at the IAT, an intensity marker of metabolic stress commonly used for training prescriptions in endurance sports [11, 12]. Since IAT did not differ between NM, SM, and FFP2, endurance training at or below IAT does not appear to have any detrimental effects on athletes when wearing a mask. Similarly, a study on steady-state exercise in non-athletes found unchanged endurance performance between SM and NM [10]. Nevertheless, in contrast to previous studies with face masks, which focused on metabolic response during exercise at a single time point [7, 10], we analyzed the submaximal performance using the BLa performance curve. Thus, it was possible to assess the metabolic response at different submaximal intensities (beyond the IAT), such as the lowered BLa at 80% peak power output with SM. The latter is likely associated with a decrease in performance as observed at maximum performance. In contrast, BLa at 20%, 40% or 60% peak power output (common training intensities in leisure sport) did not differ between NM and SM suggesting no detrimental effect on performance. Consistent with previous studies, we observed no changes in heart rate between conditions during exhaustive exercise [7–9]. In contrast, a significant increase in heart rate was observed during steady-state exercise (30-min constant exercise test) with SM, which was interpreted as a result of increased work of breathing [10]. During exhaustive exercise, only one study in healthy individuals reported an increased heart rate with SM compared with NM during a simulated hike at a comfortable pace, which was thought to be a physiological response to restricted ventilation [27]. However, limitations in statistical testing with the use of repeated measures *t*-test may challenge the external validity of this study. In contrast, heart rate did not differ in patients with established cardiovascular disease wearing an FFP1 mask during a 2-h walk compared with NM [28]. Patients with severe lung damage, such as chronic obstructive pulmonary disease, exhibit a higher heart rate after exercise (6-min walk test) when wearing an N95 respirator compared to without, which could be interpreted as a compensatory mechanism for impaired lung capacity [29]. Since a gradual decrease in diastolic blood pressure with exercise is considered normal in healthy individuals and adds no prognostic value of stress tests [30, 31], our focus was on systolic blood pressure. In line with previous studies, masks were not associated with changes in systolic blood pressure at exhaustion [7, 9].

### Limitations

The study population consisted of well-trained male endurance athletes, and thus the results cannot be generalized to females, untrained persons or patients with other diseases. Exercise testing was conducted as a short exhaustive incremental cycling test, which limits the transferability to long-term endurance exercise. Although this was the first, randomized, cross-over study to examine the effects of face masks on athlete performance, larger sample size and investigations in different sports may be needed to substantiate our findings. Due to the experimental setup, the external validity of surgical mask testing (significant leakage to the nose and ears) was limited by the fitting of the spirometry mask, which completely sealed the SM. It cannot be excluded that the sealing of the SM contributed to moisture retention within the mask, although this is influenced by several other factors such as training intensity or sweating [24, 25]. Therefore, the transferability of the investigational set-up (spirometry mask + face mask) to daily training conditions or competition of athletes remains limited. Nevertheless, dyspnea and suction of the deformed and wet SM is a phenomenon, which could be also relevant for non-athletes or patients in the presence of increasing ambient humidity and temperature (kept constant in our study).

## Conclusion

Exhaustive exercise with both SM and FFP2 was associated with reduced maximum performance in athletes. Although the athletes did not perceive increased exertion at higher intensities with mask, an unexpected abrupt exercise cessation occurred, which was described as acute dyspnea due to suction of the deformed and soaked mask (more likely to occur with SM but not with the more stable FFP2). Submaximal performance at IAT did not differ between masks and NM. For sports activities where masks are mandatory to actively contain transmission respiratory viruses, such as outdoor sports in crowded areas (i.e. city parks) or training in sports centers and gyms including participation in indoor contact sports, anaerobic intensities should be approached with caution and an FFP2 mask, which appears to be better tolerated, should be preferred over an SM.
